# Genomic sequence of a mutant strain of *Caenorhabditis elegans *with an altered recombination pattern

**DOI:** 10.1186/1471-2164-11-131

**Published:** 2010-02-23

**Authors:** Ann M Rose, Nigel J O'Neil, Mikhail Bilenky, Yaron S Butterfield, Nawar Malhis, Stephane Flibotte, Martin R Jones, Marco Marra, David L Baillie, Steven JM Jones

**Affiliations:** 1 Department of Medical Genetics, University of British Columbia, 419 - 2125 East Mall, Vancouver, BC, V6T 1Z4, Canada; 2 Genome Sciences Centre, British Columbia Cancer Research Centre, 600 West 10th Avenue, Vancouver, BC, V5Z 4E6, Canada; 3 Molecular Biology and Biochemistry, Simon Fraser University, Burnaby, BC, V5A 1S6, Canada

## Abstract

**Background:**

The original sequencing and annotation of the *Caenorhabditis elegans *genome along with recent advances in sequencing technology provide an exceptional opportunity for the genomic analysis of wild-type and mutant strains. Using the Illumina Genome Analyzer, we sequenced the entire genome of Rec-1, a strain that alters the distribution of meiotic crossovers without changing the overall frequency. Rec-1 was derived from ethylmethane sulfonate (EMS)-treated strains, one of which had a high level of transposable element mobility. Sequencing of this strain provides an opportunity to examine the consequences on the genome of altering the distribution of meiotic recombination events.

**Results:**

Using Illumina sequencing and MAQ software, 83% of the base pair sequence reads were aligned to the reference genome available at Wormbase, providing a 21-fold coverage of the genome. Using the software programs MAQ and Slider, we observed 1124 base pair differences between Rec-1 and the reference genome in Wormbase (WS190), and 441 between the mutagenized Rec-1 (BC313) and the wild-type N2 strain (VC2010). The most frequent base-substitution was G:C to A:T, 141 for the entire genome most of which were on chromosomes I or X, 55 and 31 respectively. With this data removed, no obvious pattern in the distribution of the base differences along the chromosomes was apparent. No major chromosomal rearrangements were observed, but additional insertions of transposable elements were detected. There are 11 extra copies of Tc1, and 8 of Tc2 in the Rec-1 genome, most likely the remains of past high-hopper activity in a progenitor strain.

**Conclusion:**

Our analysis of high-throughput sequencing was able to detect regions of direct repeat sequences, deletions, insertions of transposable elements, and base pair differences. A subset of sequence alterations affecting coding regions were confirmed by an independent approach using oligo array comparative genome hybridization. The major phenotype of the Rec-1 strain is an alteration in the preferred position of the meiotic recombination event with no other significant phenotypic consequences. In this study, we observed no evidence of a mutator effect at the nucleotide level attributable to the Rec-1 mutation.

## Background

*Caenorhabditis elegans *is an animal model widely used in biomedical and biological research. *C. elegans *was the first animal to have its genome completely sequenced [1] and the compiled and annotated sequence is available at WormBase http://www.wormbase.org. The ready availability of genomic sequence information along with an extensive body of knowledge about gene function in this species provides an exceptional opportunity to examine the consequences of mutational change on the composition of the genome. High-throughput sequencing of wild type [2,3] and mutant strains [4] has demonstrated the diverse benefits of examining genomic sequence. Not only is genome-wide sequencing valuable for finding the mutational basis of phenotypic change, but also for understanding evolutionary processes. Denver et al. [3] identified and characterized base-substitution mutations that arose spontaneously in 10 lines of *C. elegans*, providing us with a fuller understanding of the nature of genome-wide base-substitution events.

A question that has long been debated is the relationship of mutational patterns to biological processes such as meiotic recombination [5,6]. In *C. elegans*, the central portions of the five autosomes are relatively gene dense compared to the arms [1]. Furthermore, traditional genetic approaches using forward mutational screens to recover lethal alleles of essential genes have shown genes in the central clusters of chromosomes I and V to be more mutable to lethality than the arms [7]. However, the most striking feature of the *C. elegans *autosomes is the recombination suppression associated with the central gene clusters, [8] reviewed in [6]. In wild-type, the frequency of crossing over per length of DNA varies as much as ten-fold between the cluster and an arm of chromosome I [9], making this species an excellent model for studying the relationship between sequence variation and recombination rate.

**Figure 1:**
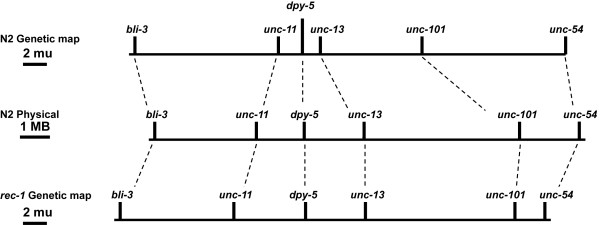
**Comparision of the Genetic and Physical Maps of chromosome I**. The top line shows the wild-type (N2) genetic map of autosome I of *C. elegans *using genetic distances measured by Zetka and Rose, 1995. Line 2 is the position of the gene markers on the physical map as annotated in WormBase http://www.wormbase.org. The bottom line is the position of markers in the Rec-1 mutant (data taken from Zetka and Rose, 1995).

The recombinational suppression of the gene clusters is eliminated in the mutant Rec-1 [10], resulting in increased crossing over in the autosomal central regions and a compensatory decrease in the arms [9]. The consequence is an altered distribution of meiotic exchange events while retaining the same overall number. In Rec-1, the genetic recombination map resembles more closely the physical length of the chromosome than it does the wild-type pattern of crossovers. The phenotype was originally identified as a recessive mutation in a strain heterozygous for morphological markers in the central cluster of chromosome I, *dpy-5(e61) unc-15(e73) *+/+ +*unc-13(e51)*. A three-fold increase in crossing over was observed in the central region of the autosomes [10]. The visible markers were eventually eliminated by recombination resulting in a wild-type appearing strain, BC313, for which the major phenotype is an altered distribution of recombination, affecting both exchange of flanking markers and apparent intragenic gene conversion [11]. There are no detrimental effects on growth, progeny number or spontaneous mutation rate. Nondisjunction of the X-chromosome is elevated somewhat, but not dramatically. The *rec-1(s180) *mutation is inherited as a Mendelian recessive, and crossover distribution is altered for the entire genome [10], including the X-chromosome, despite the fact that it has a more uniform distribution of recombination events (V. Vijayaratum and AMR, unpublished data). The consequence of the mutation is that the recombination map in Rec-1 more closely reflects the physical map than the genetic map in wild type [9] (Figure 1).

In this paper, we used the high-throughput Solexa platform (Illumina) to sequence the genome of the Rec-1 strain. This study provides the first opportunity to examine the consequences on a genome of altering the distribution of meiotic recombination events.

## Results

### Base Pair composition of Rec-1 compared to WormBase and VC2010

There were a total of 60,601,198 forty-two base pair sequence reads, of which 50,595,466 (83%) were aligned to the WormBase reference genome WS190 using MAQ software [12] with a maximum of two mismatches per read resulting in approximately 21-fold redundant sequence coverage. Base pair differences were called using both MAQ [12] and Slider [13] software.

We observed 1124 base pair differences between Rec-1 (BC313) and WormBase WS190, and 441 between Rec-1 and the wild-type strain VC2010. Fourteen of the observed differences were tested by either PCR or direct sequencing and all fourteen confirmed. In this paper, we analyzed those differences that were identified by both MAQ and Slider compared to VC2010. The canonical sequence of *C. elegans*, archived in WormBase, is valuable because it is compiled, annotated and readily accessible. The WormBase reference sequence [1] was obtained from cloned cosmids and Yacs, which for technical reasons came from different strains, and is not the genome sequence of any one existing strain. Thus, we could not experimentally determine the allelic status of *rec-1 *for the WormBase reference sequence. Since many strains of *C. elegans *carry the *s180 *allelic variant of *rec-1*, we could not simply assume the reference sequence was wild type. Thus, in this paper we examine the differences between Rec-1 and VC2010, a strain that we confirmed by measuring meiotic crossing over to be wild-type for *rec-1*.

**Figure 2:**
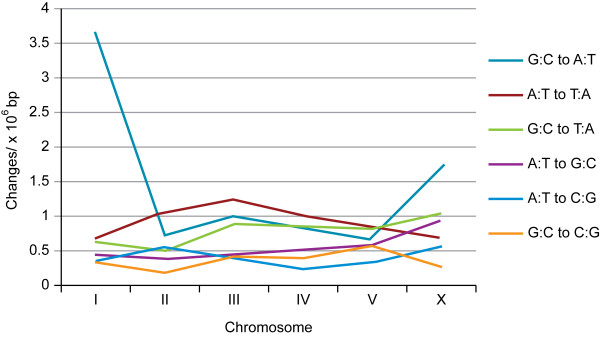
**The number of each of the nonstrand- specific types of base pair differences by chromosome**. The chromosomes are identified on the horizontal axis. The number of changes per million base pairs of aligned sequences are plotted on the vertical axis. Data from Table 1.

The *C. elegans *genome is approximately 100 million base pairs (Mbp) in size. We observed a base pair difference approximately every 225,000 bps on average. The number of base pair differences for each of the six nonstrand-specific base substitution mutation types (Table 1) per Mbp of aligned sequence was plotted for each chromosome (Figure 2). The most frequent change is G:C to A:T on chromosomes I and X, 55 and 31 respectively. Chromosome I has as many G:C to A:T substitutions as chromosomes II, III, IV and V together. Rec-1 was originally observed in strains mutagenized with EMS, a mutagen known to generate G to A changes. The gene is linked to chromosome I markers, and due to difficulty in scoring the recombination phenotype, the mutation has not been outcrossed extensively. Most likely many of the A:T differences are attributable to mutational changes retained in the Rec-1 strain. The predominance of G:C to A:T substitutions on chromosomes I and X is also reflected in the ratio of transition to transversions (Ts/Tv) (Table 1). Ignoring those two chromosomes, the Ts/Tv ratio is very close to random expectation of 0.5.

**Table 1:** Base Pair (bp) Differences between BC313 and VC2010 by Chromosome

Base-difference	I	II	III	IV	V	X	Total
G:C to A:T	55	11	14	16	14	31	141
A:T to T:A	10	16	17	17	18	12	90
G:C to T:A	9	7	12	14	17	18	77
A:T to G:C	6	6	6	9	12	17	56
A:T to C:G	5	8	5	4	7	10	39
G:C to C:G	5	3	6	7	12	5	38
Total	90	51	60	67	8	93	441
Ts/Tv	2.1	0.5	0.5	0.6	0.48	1.07	0.81

**Size in Mbp**	**15.072**	**15.279**	**13.783**	**17.494**	**20.924**	**17.719**	**100.27**
**No. aligned bp**	**14.674**	**14.878**	**13.428**	**16.857**	**20.077**	**17.394**	**97.310**

**Figure 3:**
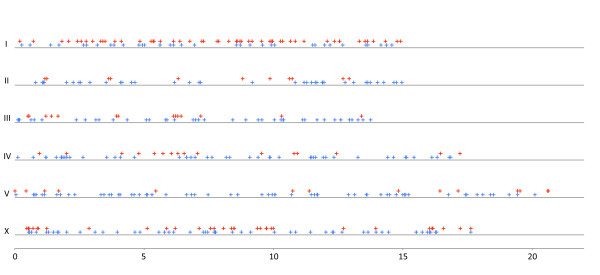
**Distribution of base pair differences between BC313 and VC2010 along the chromosomes**. Red crosses (upper) indicate the physical location of G:C to A:Ts in BC313 but not in VC2010. Blue crosses (lower) indicate the physical location of the remaining nonstrand- specific base differences. The chromosome number is shown on the X axis and the distance in Mbp along the Y axis.

Although our study is not designed to follow mutational accumulation over generations, we have analyzed the distribution of base changes along the chromosomes. Table 1 shows that the number and distribution of base pair differences on chromosome I (61%) and to a lesser extent the X-chromosome (33%) are predominantly G:C to A:T. In an attempt to separate these changes from what may be due to *de novo *mutation in Rec-1, we have plotted them separately along the chromosomes (Figure 3). Examination of the distribution of change along the chromosomes does not reveal any dramatic pattern, either for the distribution of G:C to A:T changes (upper red crosses) or for the distribution of the other types (lower blue crosses). In Denver et al. [3], after several generations of accumulated mutation in wild-type strains under relaxed selection no distinctive pattern of base substitutions was seen along the chromosomes. Neither do we see any dramatic difference in distribution that might correlate with the absence of a recombinational pattern. In an attempt to investigate the distribution numerically, we calculated the number base pair differences in the autosomal arms and in the central clusters as defined in [14] per megabasepair(Mbp). In the arms there are approximately 4.16 differences per Mbp (251/60) compared to 4.04 (97/24) in the cluster. A histogram of the number base changes per Mbp along chromosome I is shown in Additional file 1, Figure S1. When plotted this way, none of the chromosomes show any distinctive pattern (data not shown). In the absence of any detectable pattern of mutational distribution, it seems most likely that Rec-1 has had no significant affect on mutation rate.

We plotted the composition of base pair differences both for the entire genome and for the genome minus chromosome I and the X (Additional file 2, Figure S2). When chromosomes I and X are removed from the analysis, the relative frequency of the different types of base pair differences is similar to that observed by Denver et al. [3], with the exception that we see considerably fewer G:C to T:A differences.

The 441 base pair differences are shown in Additional file 3, Table S1. Ninety-five of these are in exons (22% compared to the 27% of the genome reported to be in exons [1]). Approximately half of these (51/95) were non-synonymous changes. Fifteen of the changes are in untranslated regions (UTRs), 155 in introns and the rest in intergenic regions.

### Rec-1 is neither caused by nor causes detectable chromosomal rearrangements

**Figure 4:**
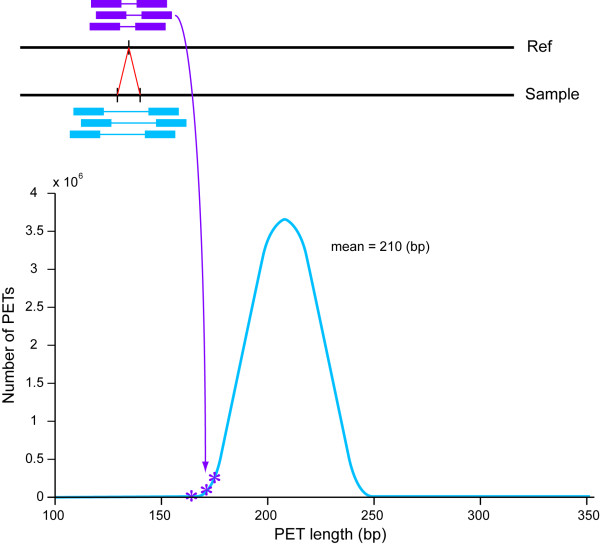
**Insertions of DNA can result in paired end tags (PETs) shorter than expected**. PETs that fall outside the normal size range can be an indication of DNA insertions. Top: In the case of a small insertion, paired end reads can cover a region in the alignment to the reference (Ref) that does not include the inserted sequence in the sample. Lower Right: Insertions in the sample are characterized by multiple PETs with a shorter than average fragment size based on the alignment.

The genome of Rec-1 was sequenced by whole genome shotgun sequencing (WGSS) using paired end tags (PETs) and aligned to the reference genome available in WormBase. Using the alignment to the WS190 reference genome, small insertions can be characterized by clusters of PETs that are shorter than the average size (Figure 4), whereas deletions in the sample can be detected by PETs that are longer. Although this is counter-intuitive to geneticists familiar with interpreting genetic maps, it is true because the sequence reads from the ends (paired end tags) of the genomic fragments are further away in the sequenced DNA if there is an insertion of unannotated material than they appear on the WormBase map (reference DNA), which lacks that insertion. In the example shown in Figure 4, the top of the figure shows the size of sequence reads aligned to the WormBase sequence. Although the inserted fragments are actually longer, they appear shorter on the reference genome. In an analogous way, deletions appear longer. Translocations will have links connecting clusters on different chromosomes with a loss of read coverage at the breakpoints. In this way, the Rec-1 sequence was analyzed for chromosomal rearrangements, insertions, deletions, inversions and translocations. No large chromosomal rearrangements were observed. Confirmational data was obtained using oligo array Comparative Genome Hybridization (aCGH). An exon-centric array design that covered the entire genome revealed no major sequence copy number changes relative to the reference DNA.

### Direct repeat sequences can appear as longer paired end reads

**Figure 5:**
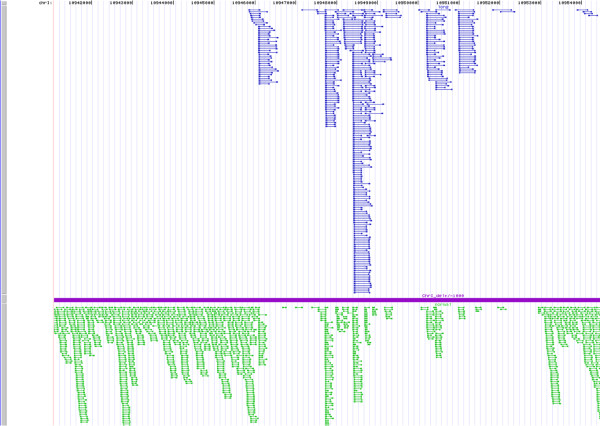
**Imperfect direct repeats can result in paired end tags (PETs) longer than expected**. PETS for bases 10,941,077-10,960,123 of chromosome I are shown. Below the line in green are the normal sized PETs. Above the line longer, potentially aberrant, PETs are shown. The longer PETs are in regions lacking normal sequence coverage. Typically, the left end of the longer tags detects unique sequence and the right end is aligned with one of a group of imperfect direct repeats producing longer than normal PETs of differing sizes.

Examination of fragment sizes in the Rec-1 strain revealed a number of paired end tags (PETs) longer than average, an indication of potential deletions. The sequence and position of these PETs were examined in detail. An example from the right arm of chromosome I is shown in Figure 5. In this case, the observed long sizes resulted from one of the paired ends aligning with an imperfect direct repeat sequence in the sample DNA. The observed longer tag length is the consequence of one of the paired ends aligning to different components of a repeat sequence. In Rec-1 as well as in other genomes analyzed, regions like this one that contained imperfect direct repeats gave poor coverage of sequence reads as is illustrated by the absence of normal size PETs. The analysis illustrates how long paired-end tags can be used to identify direct repeat sequences.

Analysis of the long PETs and alignment to the reference genome revealed the presence of two deletions in the Rec-1 strain that were not in the VC2010 wild type. One of these affected an exonic region on the X-chromosome near 11,285,000 bp and was confirmed by aCGH. The other removed approximately 100 base pairs (bp) of intergenic sequence in a region of chromosome I around 2,233,500 bp, a region of DNA not on the exon array.

### Additional transposable elements exist in Rec-1

**Figure 6:**
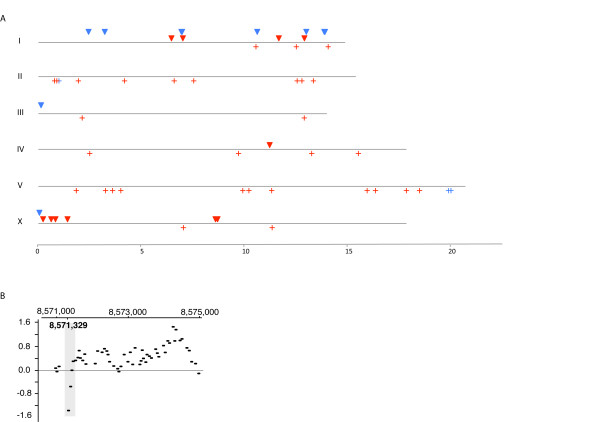
**Locations of Tc1s and Tc2s specific to Rec-1**. A. The red (Tc1) and blue (Tc2) crosses show the position of the elements in WormBase. Triangles show the positions of new full-length insertion sites for Tc1 (Red) and Tc2 (Blue). Three insertions of Tc2 on chromosome I are close together, at 3.2 Mb, 6.9 Mb, and 13.8 Mb (Table 2), and each is shown as a single triangle in the Figure. B. The aCGH data for an insertion of Tc1 into a coding region on the X-chromosome is shown.

**Figure 7:**
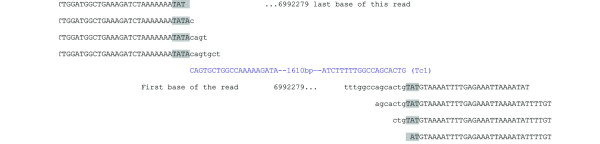
**A Tc1 insertion with the TATA at position 6992277 on chromosome I**. On the left there are four reads adjacent to the 5' end of the Tc1 sequence and on the right are four reads adjacent to 3' end. The ends of the Tc1 sequence are shown in blue. The TA is inserted at position 6,992,279. Lower case indicates read sequences that are partial Tc1's that are unmapped in the genome and shown as mismatches in the alignment. Upper case indicates read sequences that are mapped to the genome. All the reads shown are paired with another read that maps to Tc1 internal sequence.

In a progenitor strain of BC313, Tc1 was observed to actively transpose [15], although in the original CB51 strain Tc1 was apparently inactive. Blot hybridization patterns of the high-hopper strains have been published previously [15]. In this paper, we examined the number and position of the transposable elements, Tc1, Tc2, Tc3, Tc4, Tc5, Tc7 and *Cemar1 *compared to the positions reported in WormBase and reviewed by [15]. In the wild-type strain of *C. elegans *there are 30 copies of the transposable element Tc1 and four of Tc2 [16]. In the Rec-1 strain, there are 11 additional copies of Tc1, and 8 novel locations for Tc2 (Table 2; Figure 6). An example of how Tc1 insertion was analyzed is shown in Figure 7. Reads from within unique sequence paired with a read from the terminus of Tc1 identified the insertion. All the full length Tc1 and Tc2's had TA termini. We were not able to uniquely identify the progenitor Tc1 since none of the Tc1's analyzed had a unique base pair change in the portion of the element sequenced. As might be expected, most of the new insertions were in either introns or intergenic regions. One Tc1 that inserted into a coding region of a gene on the X was detectable also by aCGH. No empty sites, that is, sites vacated by Tc1, were found by searching unmapped reads for the DNA sequence TA or TATA.

**Table 2:** Sequences flanking the sites of the new Tc1 and Tc2 insertions

Chr	Position	Type	Flanking Sequences	Gene
I	6,440155	Tc1	TGCACATATA**TA**TTTGAATAGT	*snt-4 *intron

I	6,992279	Tc1	TAAAAAAATA**TA**TGTAAAATTT	C30F12.5 intron

I	11,633804	Tc1	AAAATGTACA**TA**TATGTACATA	Intergenic

I	12,872161	Tc1	TGCTCTCAAT**TA**GTACGTATCA	Intergenic

IV	11,191078	Tc1	Ambiguous insertion point	Intergenic

X	237866	Tc1	CTCCGTCAAT**TA**CAACACATGG	AC8.10

X	621137	Tc1	CATATACATA**TA**TATATATATT	*unc-96 *intron

X	827813	Tc1	CACGGAAATG**TA**GTTGGGTTCT	Intergenic

X	14,10838	Tc1	GGCTAACACA**TA**TATCCACTCA	Intergenic

X	8,571364	Tc1	GCCCAAGAAG**TA**TGTCATTGGT	*tag-279 *intron

X	8,669552	Tc1	ATCATTTAGA**TA**GATTCAAAAC	*rig-1 *intron

I	2,429485	Tc2	Ambiguous insertion point	intergenic

I	3,211994	Tc2	TTGTAGTTCA**TA**TTTAAAAAAG	*fog-1 *intron

I	3,215076	Tc2	AGATTTTAGC**TA**TTTAGAATCA	*fog-1 *intron

I	6,920634	Tc2	AAAAATGATT**TA**TCCTGATACT	*bbs-9 *intron

I	6,954054	Tc2	TGTTTACAAT**TA**GCTTTCCGAA	T10B11.8 intron

I	10,588754	Tc2	GAAACTGACC**TA**TTTTTTGTCA	*ist-1 *intron

I	12,963952	Tc2	AAAATTCATT**TA**TATAAATAAA	C47B2.2 intron

I	13,832650	Tc2	AAAAATGGGT**TA**GTTTATTATT	intergenic

I	13,867194	Tc2	Ambiguous insertion point	*taf-1 *intron

III	130778	Tc2	CAAATAGGTA**TA**TATAGTTGTT	*Nhr-280*

X	51825	Tc2	Ambiguous insertion point	intergenic

There were no new locations for Tc3, Tc4, Tc5, Tc7. These elements had positions identical to those reported in WormBase. There is an additional copy of *Cemar1 *reported in WormBase that correlates with a duplication of a portion of chromosome V, which is present in several wild-type strains, but not in VC2010 [17] or Rec-1.

### aCGH high density chip analysis agrees with the genomic sequencing

**Figure 8:**
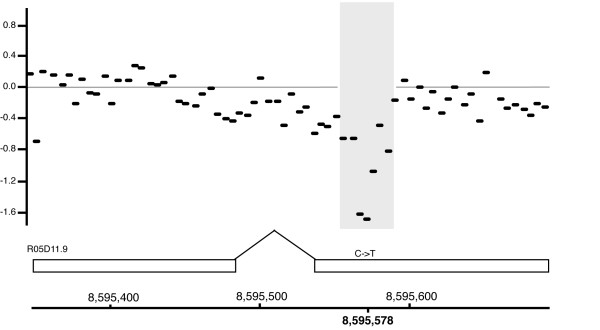
**High density aCGH**. An example of aCGH hybridization data identifying a C to T change in Rec-1. The Y-axis is the normalized log2 ratio of fluorescent intensities (Rec-1 versus reference). Each bar represents one 50 mer oligo probe on the oligo array chip. Below the plot is a schematic of the exon structure of predicted gene R05D11.9 and the position of the C-T base pair change identified at position 8,595,578 bp on chromosome I in the Rec-1 strain.

In addition to the genome-wide aCGH, a specially designed high density array was used to examine the central portion of the gene cluster of chromosome I. The array identified five base pair differences relative to the reference DNA, an example of one is shown in Figure 8. All five of these differences were also identified in the sequence analysis (see below) of the Rec-1 genomic DNA and confirmed by either restriction enzyme analysis followed by PCR or direct sequencing across the site using primers.

## Discussion

The sequence of the Rec-1 genome was obtained by whole genome shotgun sequencing (WGSS) with the Illumina Genome Analyzer and compared to both the reference genome available at WormBase http://www.wormbase.org and a laboratory wild type (VC2010) using MAQ [12] and Slider [13] software. The base pair composition of Rec-1 was more similar to the wild strain VC2010 than to the reference genome, WS190. VC2010 is a line of N2, separated from the original Brenner strain at some time in the past. There are actually two major N2 derivative lines distinguishable by the presence or absence of a duplication of a portion of the left arm of chromosome V [17]. Although the Rec-1 strain, BC313, is not directly derived from VC2010, both strains lack the chromosome V duplication. Rec-1 was originally detected in strains that along with a wild-type male strain from Brenner's original collection were transported to the BC laboratory and maintained on plates for approximately two years before being preserved by freezing in liquid nitrogen. The major detectable phenotype of Rec-1 is its alteration in crossover frequency between markers, a phenotype that is difficult and time consuming to follow through genetic crosses. For this reason, once the strain was constructed and confirmed, it was maintained without additional out-crossing.

The number, type and location of the base pair differences detected by both MAQ [12] and Slider [13] software have been analyzed for the genome. The Slider software was developed to enhance the quality of alignment possible from a low read number and improve the accuracy in base pair change prediction [13] and indeed the number of differences detected was considerably higher with the Slider software (data not shown). We confirmed the existence of a subset of the differences detected by restriction enzyme analysis of base pair differences that created new cut sites. In addition, in one region of 3 million bp chromosome I, five differences that were observed by Slider were confirmed by aCGH. aCGH has been proposed as a method for detection of single nucleotide mutations in homozygous *C. elegans *strains [18].

A large fraction of the base pair differences in Rec-1 were potentially G to A changes (141 of the 441 differences were G:C to A:T) and may represent remnants of EMS mutagenesis. Thus, we attempted to separate these from other types of changes, which may be more representative of spontaneous changes having occurred in the Rec-1 background. When plotted and compared to the mutations accumulated under relaxed selection in ten wild-type lines observed by Denver et al [3], we see a similar pattern of nonstrand-specific base substitution mutation types, with the exception that there were fewer G:C to T:A changes in Rec-1 than in their MA-lines.

The number and location of transposable elements was examined. Tc3, Tc4, Tc5, Tc7 and *Cemar1 *were unchanged. However, in addition to the ones reported in the reference genome, 11 Tc1 and 8 Tc2 were observed. These are most likely the remnants of transposition events that occurred in a progenitor strain [15]. The results emphasize the advantage over previous technologies of having the genomic sequence. We identified not only the location of the new Tc1 insertions but also the number and location of a large number of Tc2 insertions. Tc1 is 1,610 bp long and contains two 54-bp terminal inverted repeats and transposes by excision and reinsertion into target DNA containing a TA dinucleotide, leaving behind a double-strand DNA break which is repaired by the cellular machinery. Tc2 is a 2,074 base pair element that has perfect terminal inverted repeats of 24 bp and like Tc1, insertions are flanked by a TA dinucleotide at either end. It may not be obvious why Tc1 and Tc2, which are members of different transposable element superfamilies [16], would have been the two elements to have transposed in the high hopper strain [15], although both elements have been observed to transpose in Bristol Bergerac hybrids and proposed to move together possibly by a mechanism involving *mut-4 *[19]. The original strain in which mobility was first observed [15] is maintained as a frozen archive and available for further characterization with regard to aspects of Tc1 and Tc2 mobilization.

The Rec-1 strain is unique in that it alters the pattern of meiotic exchange events without affecting the total number of crossovers [9] and has little other phenotypic effects [11]. Rattray and Rose [11] investigated fitness of Rec-1 relative to wild type in a short-term experiment performed in a laboratory setting. No difference was observed under those conditions. In addition, mutational damage as measured by capture of lethal events using a genetic balancer did not differ from wild type [11]. In the present study, no major chromosomal rearrangements, which might reduce the fitness of the strain, were observed. There were however, a large number of base pair differences from wild type, and many of these were in coding regions. These changes are presumably non-detrimental based on their benign effect on the phenotype of the strain.

The genetic maps of many sexually reproducing species reveal that relative to physical distance recombination occurs more frequently in some regions than in others. Furthermore, the position of the crossover event can be influenced by a number of factors, including treatment with ionizing radiation. In Drosophila, ionizing radiation increases crossing over [20], primarily in regions of centric heterochromatin [21], a region known to have low recombination relative to the amount of DNA [22]. Similarly in *C. elegans *gamma radiation has been shown to increase crossing over across the recombination-poor central region of autosome I [23]. In yeast, DNA damaging agents have been shown to stimulate homologous recombination between ectopic repeats resulting in translocations [24]. In addition, induced double-strand breaks within dispersed small repeats can generate rearrangements resulting in genome reshaping and are a potential source for evolutionary change [25]. *C. elegans*, which is a self-fertilizing hermaphrodite with out-crossing, a relatively rare situation in nature, provides a useful model for the study of genomic characteristics as they relate to recombination and short term evolution in a self-fertilizing hermaphrodite [5,6].

In both the Denver et al. [3] analysis of ten mutation-accumulating wild-type strains and in our study of Rec-1, there is no dramatic pattern of mutational alterations along the chromosome. These results may be interesting in the context of whether or not recombination is mutagenic. Both the wild-type MA-lines, which presumably have regions of both high and low recombination, and Rec-1, in which the variation in recombination is randomized, have very similar patterns of base pair differences. The data are compatible with a model in which mutational events are independent of the meiotic recombination processes.

## Conclusion

Our analysis of high-throughput sequencing was able to detect regions of direct repeat sequences, deletions, insertions of transposable elements, and base pair differences. A subset of sequence alterations affecting coding regions were confirmed by an independent approach using oligo array comparative genome hybridization. The major phenotype of the Rec-1 strain is an alteration in the preferred position of the meiotic recombination event with no other significant phenotypic consequences. In this study, we observed no evidence of a mutator effect at the nucleotide level attributable to the Rec-1 mutation.

## Methods

### Genetic strains

Two strains of *Caenorhabditis elegans *were used in this analysis. The BC313 strain carrying the *s180 *allele of the gene *rec-1 *was constructed in 1977 and maintained frozen in liquid nitrogen. BC313 was derived from CB51 [*unc-13(e51)*], CB73 [*unc-15(e73)*] and CB61 [*dpy-5(e61*)], all of which were generated using 0.05 M ethylmethane sulfonate (EMS) in the CB laboratory of S. Brenner, Cambridge University UK [8] and transported to the BC laboratory of D. Baillie, Simon Fraser University CA where they were maintained on agar culture plates streaked with *E. coli *OP-50 at 15°C for approximately two years before being frozen. These *rec-1 *progenitor strains and the subsequent BC313 strain are estimated to have been maintained on plates for approximately 100 generations prior to sequencing.

The VC2010 strain is a wild type N2 strain subcultured in the Knock-out Consortium laboratory of D. Moerman, University of British Columbia CA from the wild-type N2 strain, VC196. It was received from the Caenorhabditis Genetics Centre in October 2008. VC2010 carries the wild-type allele of *rec-1 *and was sequenced by the Genome Sciences Centre, Vancouver CA prior to this analysis.

Brenner's wild-type N2 strain gave rise to both VC2010 and the N2 strain used in the BC313 construction, but BC313 was not derived directly from VC2010.

### aCGH

The two *C. elegans *arrays used for oligo-array comparative genome hybridization (aCGH) were designed by S. Flibotte at the Genome Sciences Centre, Vancouver CA. The whole genome array consisted of overlapping 50-mer probes targeting primarily annotated exons and micro-RNAs. Both it and the high density array were produced by NimbleGen Systems Inc. http://www.nimblegen.com. Sample preparation, hybridization and analysis was done as previously described [26].

Copy number aberrations were detected by visual inspection using the SignalMap™ browser software [NimbleGen Systems Inc. http://www.nimblegen.com.

### DNA preparation and High-Throughput Sequencing

DNA preparation for whole genome shotgun sequencing (WGSS) was done by shearing approximately 10 ug DNA for 10 min using Sonic Dismembrator 550 (cup horn, Fisher Scientific, Canada) with a power setting of "7" in pulses of 30 seconds interspersed with 30 seconds of cooling, and analyzed on a 8% PAGE gel. A 180-220 bp DNA fraction was excised and eluted from the gel slice overnight at 4°C in 300 μl of elution buffer (5:1, LoTE buffer (3 mM Tris-HCl, pH 7.5, 0.2 mM EDTA)-7.5 M ammonium acetate), and was purified using a Spin-X Filter Tube (Fisher Scientific), and by ethanol precipitation. The WGSS library was prepared using a modified paired-end protocol supplied by Illumina Inc. (USA). This involved DNA end-repair, formation of 3' A overhangs using klenow fragment (3' to 5' exo minus) and ligation to Illumina PE adapters. Adapter-ligated products were purified on Qiaquick spin columns (Qiagen) and PCR-amplified using Phusion DNA polymerase for 10 cycles using the PE primer 1.0 and 2.0 (Illumina). PCR products of the desired size range were purified using a 8% PAGE gel. DNA quality was assessed and quantified using an Agilent DNA 1000 series II assay and Nanodrop 7500 spectrophotometer (Nanodrop, USA), and DNA was subsequently diluted to 10 nM. The final concentration was confirmed using a Quant-iT dsDNA HS assay kit and Qubit fluorometer (Invitrogen). For sequencing, clusters were generated on the Illumina cluster station and paired end reads were generated using an Illumina GAII platform following the manufacturer's instructions. Image analysis, basecalling and error calibration was performed using the V1.0 Illumina Genome Analyzer analysis pipeline. The BC313 genomic sequence was aligned to the annotated sequence of *C. elegans *available at WormBase WS190 http://www.wormbase.org and compared with the sequence of the wild-type strain VC2010.

### Identification of Transposable Element Insertions

The alignment of pair-end reads was done by finding all PETs with at least one read matching 300 bp of the 5' end or 3' end of the canonical transposon sequence. The matching reads were clustered and those that had more than five reads were analyzed further. Each transposon was characterized by two clusters, one containing reads aligned to the forward strand and one containing reads aligned to reverse strand. If two clusters identified a location for a transposon that is present in the reference genome, the clusters were separated by a distance approximating the length of the transposon plus 200 bp. All novel locations has two clusters separated by about 200-300 bp. Examination of the reads flanking novel transposon locations allowed us to identify the point of insertion.

The SRA accession# is SRA009755.

## Authors' contributions

AMR, SJMJ, DLB, MM, SF, NON conceived and designed experiments. NON, MB, NM, YB and SF performed experiments. AMR, YB, MB, NON, DLB, MRJ and SJMJ analyzed data. AMR, NON, MRJ, DLB YB and SJMJ wrote the manuscript. All authors read and approved the final manuscript.

## Supplementary Material

Additional file 1 **Figure S1: Histogram of the number base differences per Mbp along chromosome I**. Data from Additional file 3, Table S1 was used to plot the number of base changes along chromosome I, revealing no obvious difference for different regions of the chromosome. Click here for file

Additional file 2 **Figure S2: The total number of each of the nonstrand- specific types of base pair differences**. Blue bars indicate the total base pair differences between Rec-1 and VC2010 for the genome. Red bars indicate the differences for chromosomes II, III, IV and V summed together. Click here for file

Additional file 3 **Table S1: Base Differences between BC313 and VC2010**. All the base differences identified by both MAQ and Slider are listed and annotated. Each difference is identified by a unique 'h' allele designation. Click here for file
