# Supplementary figures and images for: Genomic sequence of a mutant strain of Caenorhabditis elegans with an altered recombination pattern
Source: BMC Genomics. 2010 Feb 23;11:131. doi: 10.1186/1471-2164-11-131 (PMC2837035; doi:10.1186/1471-2164-11-131)

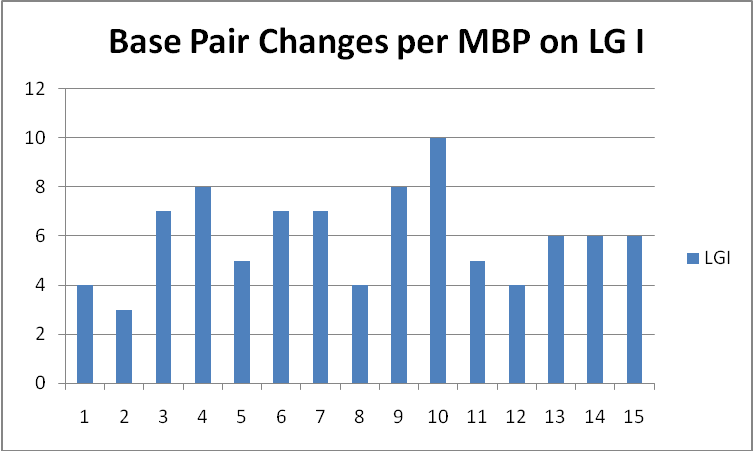

Supplement: Additional file 1 — Figure S1: Histogram of the number base differences per Mbp along chromosome I. Data from Additional file 3, Table S1 was used to plot the number of base changes along chromosome I, revealing no obvious difference for different regions of the chromosome. [file 1471-2164-11-131-S1.DOC]

# Base Pair Differences

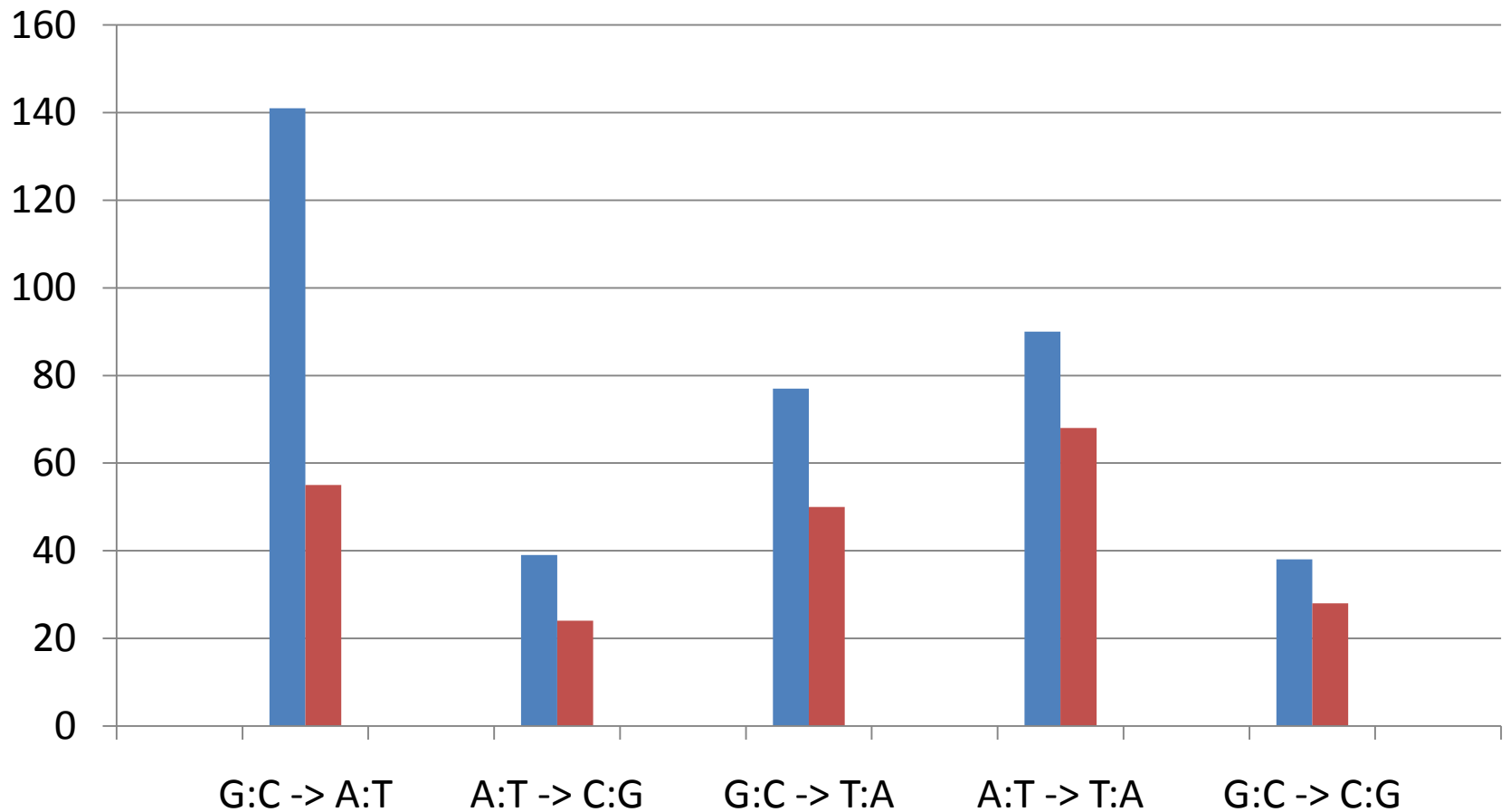

Supplement: Additional file 2 — Figure S2: The total number of each of the nonstrand- specific types of base pair differences. Blue bars indicate the total base pair differences between Rec-1 and VC2010 for the genome. Red bars indicate the differences for chromosomes II, III, IV and V summed together. [file 1471-2164-11-131-S2.PDF]
